# *Antrodia cinnamomea*, a Treasured Medicinal Mushroom, Induces Growth Arrest in Breast Cancer Cells, T47D Cells: New Mechanisms Emerge

**DOI:** 10.3390/ijms20040833

**Published:** 2019-02-15

**Authors:** Yu-Cheng Chen, Yi-Chang Liu, Mohamed El-Shazly, Tung-Ying Wu, Jan-Gowth Chang, Yang-Chang Wu

**Affiliations:** 1 The Ph.D. Program for Cancer Biology and Drug Discovery, China Medical University and Academia, Sinica, Taichung 404, Taiwan; j520c@hotmail.com; 2 Division of Hematology-Oncology, Department of Internal Medicine, Kaohsiung Medical University, Hospital, Kaohsiung 807, Taiwan; ycliu@cc.kmu.edu.tw; 3 Department of Internal Medicine, Faculty of Medicine, College of Medicine, Kaohsiung Medical University, Kaohsiung 807, Taiwan; 4 Department of Pharmacognosy, Faculty of Pharmacy, Ain-Shams University, Organization of African Unity Street, Abassia, Cairo 11566, Egypt; mohamed.elshazly@pharma.asu.edu.eg; 5 Department of Pharmaceutical Biology, Faculty of Pharmacy and Biotechnology, German University in Cairo, Cairo 11432, Egypt; 6 Chinese Medicine Research and Development Center, China Medical University Hospital, Taichung 404, Taiwan; kuma0401@gmail.com; 7 School of Medicine, China Medical University, Taichung 404, Taiwan; 8 Department of Laboratory Medicine, China Medical University Hospital, Taichung 404, Taiwan; 9 Center for Precision Medicine, China Medical University Hospital, Taichung 404, Taiwan; 10 Graduate Institute of Natural Products, College of Pharmacy, Kaohsiung Medical University, Kaohsiung 807, Taiwan; 11 Department of Medical Research, Kaohsiung Medical University Hospital, Kaohsiung 807, Taiwan

**Keywords:** *Antrodia cinnamomea*, breast cancer, endoplasmic reticulum stress, histone deacetylases

## Abstract

Reported cases of breast cancer have skyrocketed in the last decades with recent advances in examination techniques. Brest cancer has become the second leading cause of mortality among women worldwide, urging the scientific community to develop or find new drugs from natural sources with potent activity and a reasonable safety profile to tackle this ailment. *Antrodia cinnamomea* (AC) is a treasured medicinal fungus which has attracted attention due to its potent hepatoprotective and cytotoxic activities. We evaluated the antiproliferative activity of the ethanol extract of artificially cultured AC (EEAC) on breast cancer cells (T47D cells) in vivo and in vitro. Ethanol extract of artificially cultured AC inhibited T47D cells’ proliferation mediated by cell cycle arrest at G1 phase as well induced autophagy. Immunoblotting assay confirmed that EEAC not only decreased the expression of the cell-cycle-related proteins but also increased the expression of transcription factor FOXO1, autophagic marker LC3 II, and p62. Ethanol extract of artificially cultured AC mediated endoplasmic reticulum stress by promoting the expression of IRE1 (inositol-requiring enzyme 1α), GRP78/Bip (glucose regulating protein 78), and CHOP (C/EBP homologous protein). Apart from previous studies, HDACs (histone deacetylases) activity was inhibited as demonstrated by a cell-free system, immunoblotting, and immunofluorescence assays following EEAC treatment. The in vivo studies demonstrated that EEAC decreased tumor volume and inhibited tumor growth without any significant side effects. High performance liquid chromatography profile demonstrated similar triterpenoids compared to the profile of wild AC ethanol extract. The multiple targets of EEAC on breast cancer cells suggested that this extract may be developed as a potential dietary supplement targeting this debilitating disease.

## 1. Introduction

Breast cancer is the second leading cause of cancer-associated mortalities among woman in developed eastern and western countries [1,2]. There are three types of breast cancer including estrogen receptor (ER) positive cancer, human epidermal growth factor two positive (HER2) cancer as well as triple negative breast cancer (TNBC) which is negative for ER, PR (progesterone receptor), and HER2 [3]. In the last few decades, many molecular targets in breast cancer were discovered aiming to treat this disease based on tumor establishment and receptors status. Several chemotherapeutic agents were developed targeting specific molecular targets including capecitabine as an antimetabolite; docetaxel and paclitaxel as antimitotic; trastuzumab as immunotherapy; and tamoxifen as hormone therapy [4]. Recently, multiple therapies were combined aiming to improve prognosis and overcome resistance. However, breast cancer remains a deadly disease resulting in thousands of deaths every year [5]. Thus, there is a critical need to develop novel therapeutic candidates for breast cancer patients.

Aiming to find new therapeutic agents targeting breast cancer, scientists have tried to understand the effect of the epigenetic modification on this type of cancer. Epigenetic modification affects gene expression without changing the DNA sequence. There are many epigenetic modification approaches including DNA methylation, small RNAs, and histone modification [6]. One of the histone modification approaches is done through histone acetyl-transferases (HATs) and histone deacetylases (HDAC). These enzymes control gene expression at the N-terminal lysine residues leading to histone acetylation and deacetylation. These processes result in chromatin de-condensation or condensation which stimulate or inhibit genes expression [7]. There are four identified classes of HDACs including Class I (HDAC 1, 2, 3, and 8), Class IIa (HDAC 4, 5, 7, and 9), Class IIb (HDAC 6 and 10), Class III (HDAC11) as well as Class IV (sirtuins 1-7) [8]. Previous studies indicated that HDAC 1 was highly expressed in hormone receptor-positive breast tumors, while HDAC 2, 3, and 4 were strongly expressed in the more aggressive breast tumors [9,10,11,12]. Therefore, inhibiting HDACs emerged as an attractive technique to cure breast cancer patients.

Other molecular targets were also studied in the war against cancer including endoplasmic reticulum (ER), which is an essential cellular organelle that maintains cellular function and proliferation. It is the center of proteins synthesis, folding, and quality control [13]. It also modulates and stores calcium ions. In certain cases, the capacity of ER gets overloaded resulting in ER stress and unfolded protein response (UPR). These effects are modulated by pancreatic endoplasmic reticulum kinase (PERK), inositol-requiring enzyme 1α (IRE1α), and activating transcription factor 6 (ATF6) [14]. The activation of PERK, ATF6, and IRE1α are modulated by GRP78/Bip (glucose regulating protein 78) which acts as a monitor of ER stress and affects cellular survival [15]. If UPR response continues, it causes cell growth inhibition or cellular apoptosis [16]. The relationship between UPR and cellular death renders UPR as an attractive target for synthetic and natural anticancer agents. 

Natural products isolated from Chinese herbs are important sources for biologically active agents targeting human ailments especially cancers due to their capacity to induce apoptosis, suppress angiogenesis and enhance the chemotherapeutic activity of synthetic drugs [17]. Among these Chinese herbal treasures is a valued and rare medicinal mushroom, *Antrodia cinnamomea* (AC). It is a unique medicinal fungus which is endogenous to Taiwan. Its fruiting bodies were used by aboriginal tribes as a decoction or chewing material for the treatment of discomfort caused by excessive alcohol intake [18]. Recent studies indicated that AC extract exhibited hepatoprotective activity against hepatotoxicity induced by alcohol consumption [19]. It also protected the liver against fibrosis induced by CCl_4_ [20]. The extract exhibited cytotoxic activity against liver cancer cells through the inhibition of Bcl_2_ [21] and modulated calcium-calpain-mitochondria signaling pathway [22]. When tested against breast cancer cells, AC extract inhibited COX-2 expression in MDA-MB-231 cells and activated caspase-3 in MCF-7 cells as well as it induced DNA damage in T47D cells resulting in cellular apoptosis [23,24,25]. *Antrodia cinnamomea* and its active constituents were subjected to an extensive investigation to reveal their therapeutic potential applications as dietary supplements and functional foods [26]. In the current study, we investigated the effect of ethanol extract of dish-cultured AC (EEAC) on ER stress and HDACs inhibition which has barely been investigated in previous literature.

## 2. Results

### 2.1. EEAC Exhibits Cytotoxic Activity against Human Breast Cancer Cell Line T47D without Induction of Apoptosis

**Figure 1:**
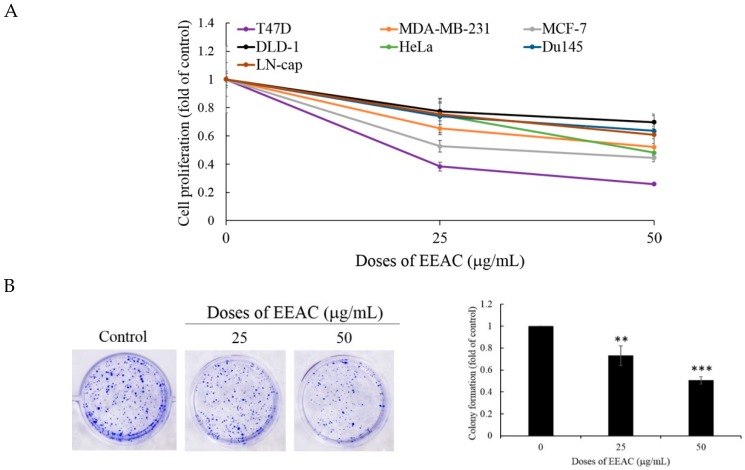

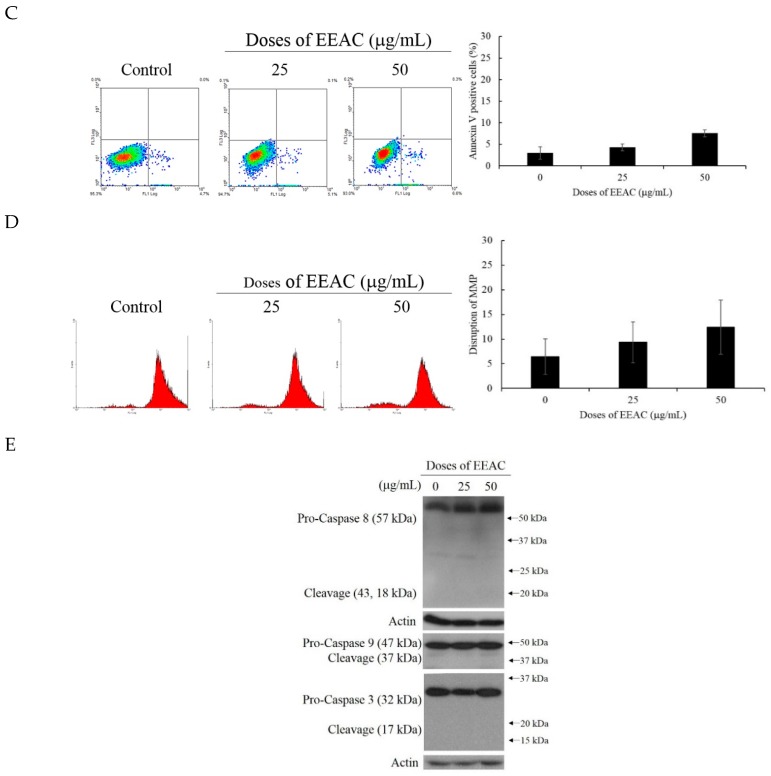
Ethanol extract of artificially cultured AC (EEAC) (25 and 50 μg/mL) inhibited cancer cell proliferation without the induction of cellular apoptosis and disruption of mitochondrial membrane potential. (**A**) Human cancer cell lines were treated with EEAC and incubated for 72 h and assessed by MTT assay; (**B**) effect of EEAC on colony formation in T47D cells; T47D cells were treated with EEAC, incubated for 48 h, and stained with (**C**) Annexin V and propidium iodide and (**D**) Rhodamine 123; (**E**) the expression of pro-apoptosis protein caspases-3, -8, and -9 was determined by Western blot assay. Actin was used as the loading control. All results are presented as mean ± SD of at least three experiments, ** *p* < 0.01; *** *p* < 0.001.

To fully understand the cytotoxic potential of EEAC, we screened its anti-cell proliferative activity with several cancer cell lines including colon (DLD-1), cervical (Hela), prostate (Du145 and LN-cap) as well as breast (T47D, MCF-7, and MDA-MB-231) for 72 h. Breast cancer cell line T47D was the most sensitive cell line with the IC_50_ value 13 μg/mL as demonstrated by the MTT assay. To determine EEAC’s long-term anti-proliferative activity, the colony formation assay was used. Our results demonstrated cell growth inhibition of T47D cells to EEAC (25 and 50 μg/mL) treatment resulting in a 27% and 50% decrease of colony formation, respectively (Figure 1A,B). The potent anti-cell proliferative activity prompted us to determine the cytotoxic mechanism of EEAC using the T47D cell line. First, we investigated whether the anti-cell proliferative activity of EEAC was associated with apoptosis induction using the annexin-V-FITC and propidium iodide (PI) assay. We also used rhodamine 123 staining which stains living cells’ mitochondria and is used to determine mitochondrial membrane potential. As shown in Figure 1C,D, treating T47D cells with EEAC (25 and 50 μg/mL) for 48 h did not induce cell apoptosis nor disrupt mitochondrial membrane potential. In order to further confirm that the anti-cell proliferative activity of EEAC was not caused by the induction of cellular apoptosis, we evaluated the expression of pro-apoptotic proteins including caspases-3, -8, and -9. The treatment of T47D cells with EEAC (25 and 50 μg/mL) did not change the expression of caspases-3, -8, and -9 (Figure 1E). Our results indicated that EEAC significantly inhibited T47D cells’ proliferation in a dose-dependent manner without affecting the extrinsic and intrinsic apoptotic pathway and mitochondrial membrane potential.

### 2.2. Effect of EEAC on Cell Cycle and Autophagy

**Figure 2:**
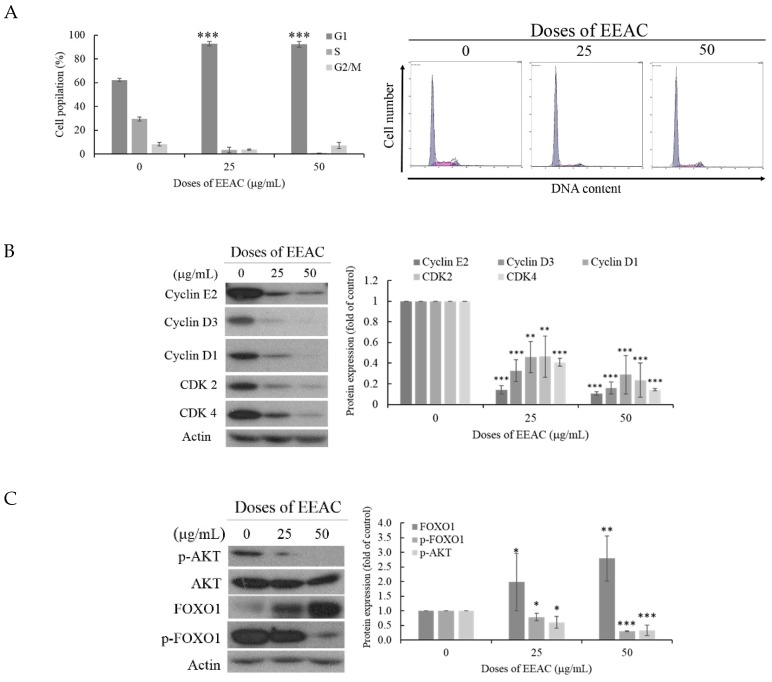

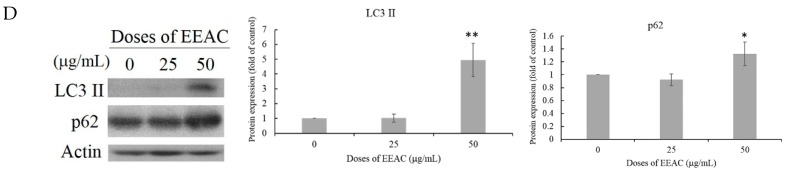
Effect of EEAC on cell cycle progression and autophagy induction. T47D cells were treated with EEAC (25 and 50 μg/mL) for 48 h. (**A**) Determination of cell cycle progression using flow cytometry and quantification was done by MultiCycle software. (**B**) Expression of G1 phase-related proteins cyclin E2, cyclin D3, and cyclin D1, CDK2, and CDK4 was determined by Western blot assay. (**C**) Expression of AKT/FOXO1 signaling pathway proteins was determined by Western blot assay. (**D**) Expression of autophagy-related proteins LC3 II and p62 was determined by Western blot assay. Actin was used as the loading control. All the results are presented as mean ± SD of at least three experiments, * *p* < 0.05; ** *p* < 0.01; *** *p* < 0.001.

As demonstrated in Figure 1, EEAC significantly inhibited T47D cells growth without the induction of cellular apoptosis. To clarify the anti-proliferative effect of EEAC, we evaluated the effect of EEAC on cell cycle population and autophagy. As demonstrated in Figure 2A, EEAC treatment significantly increased the percentage of T47D cells in G1 phase. The percentage of T47D cells in G1 phase increased from 62.3% to 92.8% and 92.3% when treated with EEAC (25 and 50 μg/mL) for 48 h, respectively. We also determined the expression of cell-cycle-regulator-related proteins using a Western blotting assay. The treatment of T47D cells with EEAC (25 and 50 μg/mL) for 48 h significantly decreased the expression of the cell cycle related proteins. The expression of cyclin E2, cyclin D3 and cyclin D1, CDK2, and CDK4 were reduced 85%, 67%, 54%, 53%, and 59% with 25 μg/mL of EEAC and 90%, 84%, 71%, 76% and 86% with 50 μg/mL of EEAC, respectively (Figure 2B). It has been suggested that the AKT/FOXO1 signaling pathway plays an important role in several cancers especially in breast, thyroid and cervical cancers. The depletion of AKT triggers FOXO1 activation mediated cell cycle arrest or cell apoptosis, however, the phosphorylation of FOXO1 results in the loss of tumor suppressor function [27]. The expression of AKT/FOXO1 signaling pathway was measured by Western blotting assay after treatment with EEAC (25 and 50 μg/mL) for 48 h. As shown in Figure 2C, the expression of phosphorylated AKT and FOXO1 was significantly suppressed by 40% and 21% in response to 25 μg/mL of EEAC as well as 67% and 70% with the treatment of 50 μg/mL of EEAC, respectively. The expression of FOXO1 was increased by 2 and 2.7-folds with EEAC 25 and 50 μg/mL treatments, respectively. Another mechanism which is related to cellular proliferation is autophagy. Studies indicated that the inhibition of CDK2 and CDK4 enhanced autophagy in breast cancer cells [28]. To further explore the effect of EEAC on autophagy, we determined the expression of autophagy markers LC3 II and p62 using Western blotting assay. As shown in Figure 2D, the expression of LC3 II and p62 increased by 5- and 1.3-fold in response to the treatment of 50 μg/mL of EEAC, respectively. These results indicated that EEAC modulated cellular proliferation through the inhibition of cell cycle progress at G1 phase and affected AKT/FOXO1 pathway at both concentrations (25 and 50 μg/mL) but the induction of autophagy was observed only at 50 μg/mL.

### 2.3. Effect of EEAC on the Expression of Endoplasmic Reticulum (ER) Stress-Related Proteins in T47D Cells

**Figure 3:**
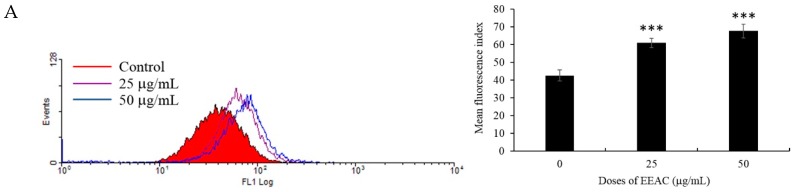

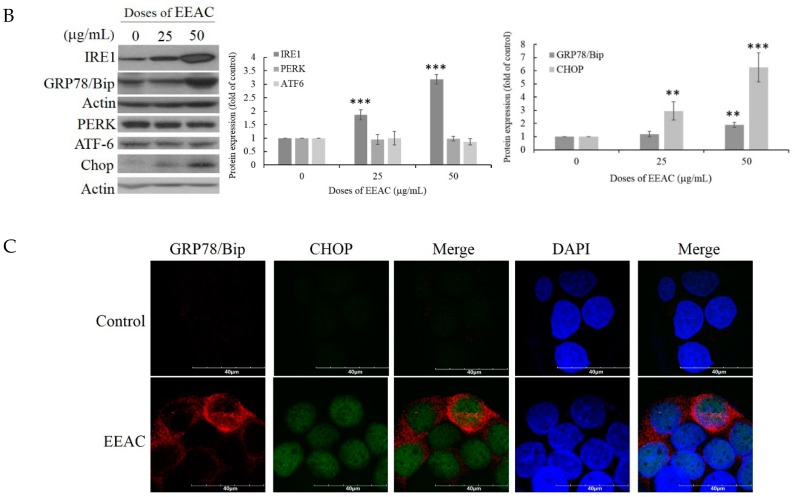
Effect of EEAC on endoplasmic-reticulum (ER). T47D cells were treated with EEAC (25 and 50 μg/mL) for 48 h. (**A**) Cells were collected and stained with Fluo-3 AM for the determination of Ca^2+^ concentration and analyzed using flow cytometry. (**B**) Expression of ER stress sensor proteins IRE1 (inositol-requiring enzyme 1α), PERK (pancreatic endoplasmic reticulum kinase), and ATF-6 (activating transcription factor 6), as well as ER stress marker GRP78/Bip and CCAAT-enhancer-binding protein homologous protein (CHOP), was determined by Western blot assay. Actin was used as the loading control. (**C**) Confocal microscopy image of GRP78/Bip (red) and CHOP (green) stained T47D cells were treated with EEAC (50 μg/mL) for 48 h. Cells were counterstained with DAPI to label cells nuclei (blue). All the results are presented as mean ± SD of at least three experiments, ** *p* < 0.01; *** *p* < 0.001.

Studies indicated that the induction of ER stress interfered with cell cycle progress [29], autophagy, apoptosis, and chemoresistance in human breast cancer cells [30]. Endoplasmic reticulum stores calcium and controls cellular calcium concentration [31]. Under ER stress, calcium is released from the ER to the cytoplasm [32]. Due to the significant role of ER in cellular homeostasis, we investigated if ER stress is involved in EEAC-induced cell cycle arrest and induction of autophagy. First, we examined the effect of EEAC (25 and 50 μg/mL) treatment for 48 h on the intracellular Ca^2+^ concentration using Fluo-3 AM and flow cytometry assay. As shown in Figure 3A, Ca^2+^ concentration increased by 1.4- and 1.6-fold compared with the control group suggesting that EEAC may trigger ER stress. To further confirm if EEAC causes ER stress, Western blotting assay was used to determine the expression of UPR proteins which are activated when ER functions are overloaded. As shown in Figure 3B, the expression of ER stress sensor IRE1 significantly increased by 1.9- and 3.1-fold but not the expression of PERK and ATF-6 compared with the control group after treatment with EEAC (25 and 50 μg/mL) for 48 h. Recent studies indicated that GRP78/Bip pathway keeps stress sensors in an inactive state [33]. When the unfolded proteins in ER lumen are overloaded, GRP78/Bip detaches from these sensors activating UPR mediated downstream protein CHOP (CCAAT-enhancer-binding protein homologous protein). Our results showed that EEAC treatment promoted the expression of ER stress markers CHOP and GRP78/Bip as demonstrated by Western blotting assay (Figure 3B). In a previous study, CHOP accumulated in the nucleus was suggested to control cell cycle progression [34]. Our immunofluorescence results supported previous reports and showed that EEAC treatment stimulated CHOP expression resulting in its accumulation in the nucleus. Ethanol extract of artificially cultured AC also promoted GRP78/Bip expression (Figure 3C). Our results suggested that EEAC triggered UPR mediated ER stress by IRE1 activation.

### 2.4. Effect of EEAC on Histone Deacetylase Activity in T47D Cells

**Figure 4:**
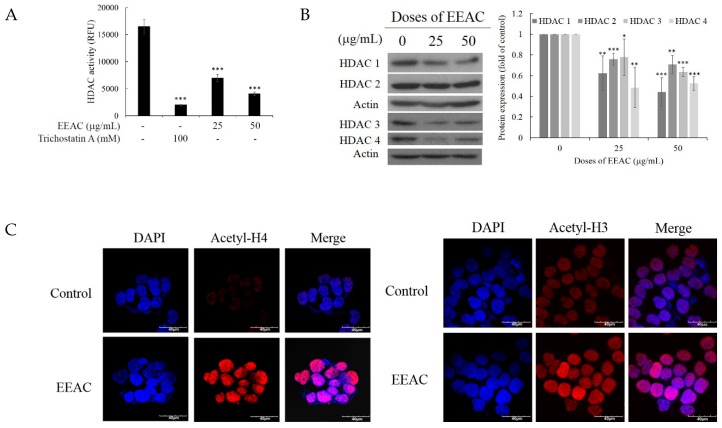
Effect of EEAC on histone deacetylase activity. (**A**) The effect of EEAC on HDAC mediated deacetylation in the cell-free system. (**B**) T47D cells were treated with EEAC (25 and 50 μg/mL) for 48 h and the expression of HDACs 1, 2, 3, and 4 was determined by Western blot assay. Actin was used as the loading control. (**C**) Confocal microscopy image of acetyl-H3 and acetyl-H4 stained T47D cells treated with EEAC (50 μg/mL) for 48 h. Cells were counterstained with DAPI to label cells nuclei (blue). All the results are presented as mean ± SD of at least three experiments, * *p* < 0.05; ** *p* < 0.01; *** *p* < 0.001.

To evaluate if EEAC modulates epigenetics, the cell-free HDAC colorimetric assay was used to screen the effect of EEAC on HDAC activity. An HDAC inhibitor, trichostatin A, served as the control. As shown in Figure 4A, EEAC inhibited HDAC activity in a dose-dependent manner. The expression of HDACs was further examined with Western blotting assay. Ethanol extract of artificially cultured AC (25 μg/mL) inhibited HDACs expression including HDAC 1, HDAC 2, HDAC 3, and HDAC 4 showing 38%, 24%, 22%, and 51% reduction, respectively. The use of EEAC (50 μg/mL) resulted in more reduction of HDAC 1 (56%), HDAC 2 (29%), HDAC 3 (36%), and HDAC 4 (48%) as shown in Figure 4B. The immunofluorescence results further confirmed that EEAC (50 μg/mL) inhibited HDACs activity resulting in the acetylation of histones H3 and H4 (Figure 4C; Figure S1). These results indicated that EEAC modulated epigenetics through the inhibition of HDACs activity mediated histone acetylation.

### 2.5. Effect of EEAC on the Growth of Human Breast Tumor in in Vivo Xenograft Animal Model

**Figure 5:**
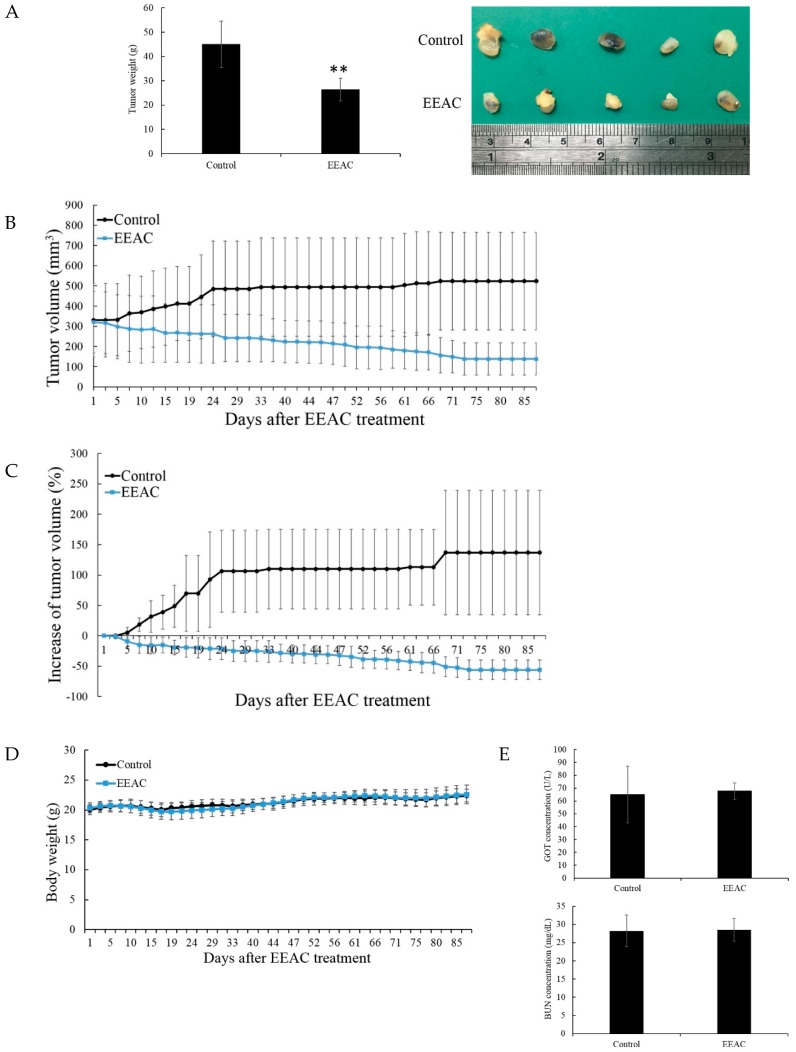

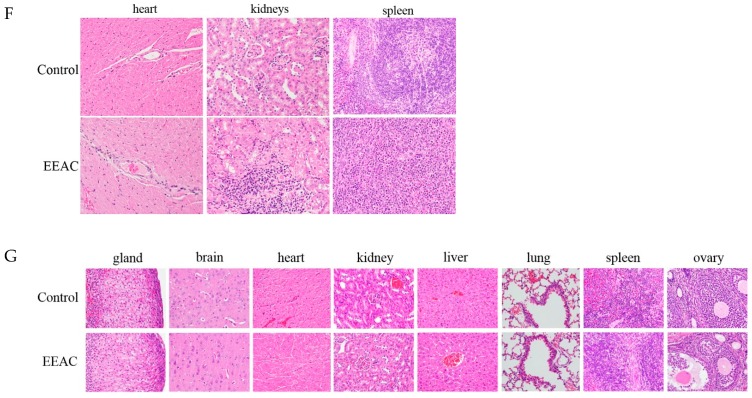
Anti-tumor effect of EEAC on tumor growth in vivo animal model. Tumor-bearing nude mice were treated with DMSO or EEAC (100 μg/g) for 87 days (each group contained five mice). (**A**) Photo of tumors (left) and the histogram results of tumor weights (right). (**B**) Tumor volumes were measured every other day. (**C**) Effect of EEAC on the increase of tumor volume. (**D**) Nude mice body weights were measured every other day. (**E**) Chemical plasma profiles were determined with FUJIFILM colorimetric analyzer (DRI-Chem 3000). (**F**,**G**) Tissue sections from nude mice organs were stained with hematoxylin and eosin (400×). All the results are presented as mean ± SD, ** *p* < 0.01.

Our in vitro results indicated that EEAC inhibited T47D cells’ proliferation. To further evaluate the anti-tumor activity of EEAC, we used a xenograft nude mice animal model inoculated with T47D cells. As shown in Figure 5A, EEAC decreased tumor weight from 44.9 ± 9.6 to 26.3 ± 4.6 g resulting in 41% reduction compared with the control after EEAC (100 μg/g) treatment for 87 days. Our results indicated that EEAC significantly decreased tumor volume starting from day 19. The tumor volume was 523 ± 242 mm^3^ in the control group while 138 ± 80 mm^3^ in EEAC treated group showing 73% reduction at day 87 (Figure 5B). Our results also indicated that the increase of tumor volumes was significantly different at day 5, and there was 193% decrease at day 87 compared with the control group showing 56% reduction compared with the starting volume (Figure 5C). No significant differences in the functions of liver and kidney were noticed as well as no histological differences were detected in the heart, kidney, and spleen and body weight in EEAC treated group (Figure 5D–F). To further confirm the absence of significant side effects with EEAC treatment, the 30-day subacute toxicity study of EEAC in nude mice was evaluated. Ethanol extract of artificially cultured AC (250 μg/g) and osmosis water (control group) administered orally to nude mice for 30 consecutive days. During the experiment period, no abnormal changes were showed in the clinical picture nor body weights and all the mice were survived to the end. Histopathological examinations between the EEAC and control groups provided a primary safety profile (Figure 5G). 

### 2.6. HPLC Profiles of AC Ethanol Extract of Wild Fruiting Bodies and EEAC

**Figure 6:**
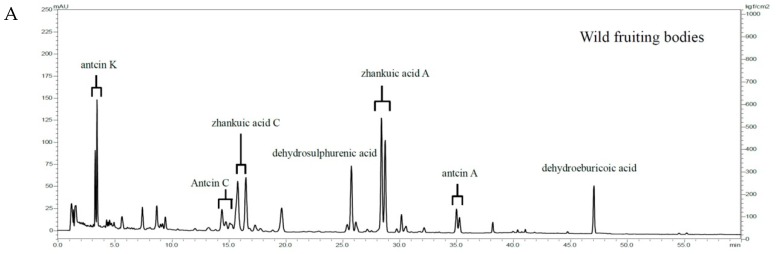

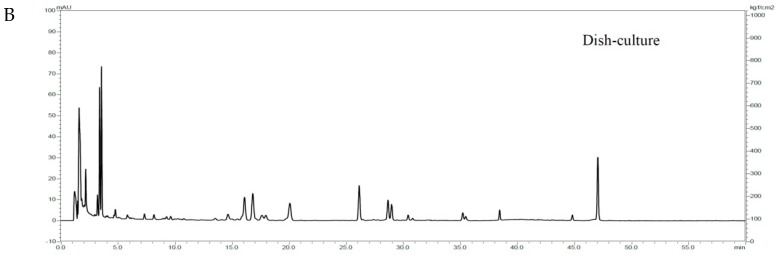
HPLC profiles of AC major triterpenoids of the ethanol extract of (**A**) wild fruiting bodies and (**B**) dish-cultured product (EEAC).

Triterpenoids are considered the major active components of AC which are responsible for its pharmacological and therapeutic effects [35]. HPLC profiles of AC major triterpenoids of the ethanol extract of wild fruiting bodies and EEAC were evaluated (Figure 6A,B). Our results showed that EEAC contained the same major triterpenoids compared to the wild AC extract. The results showed that EEAC could be considered as one of the alternative sources of AC that effectively reduces tumor growth and exhibits an anti-tumorigenic effect in vivo without any significant side effect.

## 3. Discussion

Medicinal mushrooms attracted attention in the last few decades as potential chemopreventive agents with high safety profile [36]. Collection from nature was the only source of these natural treasures until recent advances in cultivation methods. Advanced cultivation methods offered a steady and reliable source of medicinal mushrooms. Scientists extensively investigated mushroom culturing conditions and tried to mimic the conditions of natural mushroom habitats aiming to produce cultured products with HPLC profiles similar to those of natural mushrooms. However, strain type, media conditions, and culturing method all affect the HPLC profile of the mushroom [37,38]. *Antrodia cinnamomea* (AC), a Taiwanese endemic species, has been used as a chemopreventive mushroom in folk medicine for centuries [39]. The slow growth rate and high market demand of wild AC fruiting bodies encouraged research groups and food dietary companies to develop new cultivation techniques to provide steady supply at reasonable prices. In general, there are five common sources of AC including the wild-type, submerged fermentation, solid support culture, cutting wood culture, and dish culture. The chemical profiles of wild and cultivated ACs’ major components may be similar; however, their biological functions are vastly different as their activity depends on their overall spectrum of ingredients. Our EEAC showed a triterpenoids profile similar to the unique and characteristic profile of the wild fruiting bodies (Figure 6A,B) using our previously reported method that standardized triterpenoids in AC (Japanese Patent, No. 6325018).

Previous studies showed that AC modulated cell cycle progression in hepatocellular carcinoma [40], leukemia [41], colorectal cancer [42], and lung cancer [43]. In T47D breast cancer cells, Shang et al. [25] indicated that AC inhibited the expression of cell cycle-related proteins by the modulation of the PI3K/AKT/mTOR signaling pathway and induction of cell apoptosis [25]. In the current study, we further demonstrated that EEAC interrupted cell cycle at G1 phase and decreased the expression of G1 phase-related proteins without induction of apoptosis. We also found for the first time that EEAC modulated the AKT/FOXO1 signaling pathway (Figure 1 and Figure 2). FOXO1 is a transcription factor that plays important roles in the regulation of cell cycle progression. Previous reports also demonstrated that the loss-of-function of FOXO1 could lead to the prostate, breast, and thymic tumors formation [44,45]. Another important factor which modulates cellular proliferation is autophagy. Recent reports indicated that AC induced autophagic cell death in colorectal cancer cells and triple-negative breast cancer (MDA-MB-231) cells [46,47]. Our results demonstrated that EEAC (50 μg/mL) induced autophagic marker LC 3II and p62 protein suggesting that autophagy also plays a central role in EEAC inhibition of T47D cells’ proliferation (Figure 2D).

The endoplasmic reticulum is an important cellular organelle, which modulates protein folding, drugs detoxification, and calcium storage to overcome many cellular stresses. Its role in tumor growth introduced this organelle as an attractive target for cancer therapy [48]. In a previous study, AC protected a mouse pancreas cell line, Beta-TC-6 cells, from apoptosis induced by thapsigargin through the reduction of IRE1 expression showing antidiabetic activity [49]. However, there is limited information on AC’s regulatory effect on ER stress in cancer cells. In human colorectal cancer, AC increased CHOP expression [46]. In our study, we revealed that EEAC induced ER stress by activating ER stress sensor IRE1 and mediated CHOP expression in cells’ nuclei. However, it did not affect ATF-6 nor PERK (Figure 3). 

Histone acetylation is one of the post-translation modifications that is regulated by histone acetylation and histone deacetylation. It was suggested the inhibition of HDACs is an appealing target for cancer treatment due to the observed dysregulation of its functions in many cancers. Abexinostat, an HDAC inhibitor (HDACi), is now in phase II clinical trials for the treatment of breast cancer [50]. Several protocols were introduced for the use of HDACi in combination with other chemotherapeutic treatments including radiotherapy [51], topoisomerase inhibitors [52], platinum-based chemotherapeutic [53], and hormonal therapy [54]. From our previous study, we demonstrated that the wild fruiting bodies of AC induced cellular apoptosis through HDAC1 hypoacetylation in leukemia HL60 cells [55]. In the current study, we demonstrated that EEAC significantly inhibited HDAC1, HDAC2, HDAC3, and HDAC4 expression mediated acetyl-histones H3 and H4 activation in T47D cells (Figure 4). Our results provided further insight into the inhibitory activity of EEAC on HDACs in breast cancer T47D cells.

In conclusion, our HPLC profiles indicated that our unique culture method successfully produced a cultured product with similar major active triterpenoids observed in the profile of wild fruiting bodies extract. Our results also confirmed that the treatment of T47D cells with EEAC induced ER stress through IRE1 activation mediated anticancer protein CHOP expression as well as HDACs inhibition mediated acetyl-histones H3 and H4 activation, leading to cell cycle arrest at G1 phase and autophagy induction without causing cell apoptosis. An in vivo animal model also confirmed that EEAC can act as a potential anti-tumor agent without any significant side effects (Figure 5). Our results shed light on EEAC’s inhibitory effect on cellular proliferation through cell cycle arrest and induction of autophagy mediated by ER stress and HDACs inhibition. 

## 4. Materials and Methods

### 4.1. Bioassay Materials

All human cancer cell lines were purchased from the American Type Culture Collection (Manassas, VA, USA). RPMI 1640 medium, fetal bovine serum, trypan blue, anti-anti were purchased from Gibco, all other chemicals were purchased from Sigma–Aldrich (St. Louis, MO, USA). Antibodies against caspases-3 (1:1000; Rabbit IgG), -8 (1:1000; Mouse IgG), and -9 (1:1000; Rabbit IgG), FOXO 1 (1:500; Rabbit IgG), phosphorylated-FOXO1 (1:1000; Rabbit IgG), AKT (1:1000; Rabbit IgG), Cyclins E2 and D1 (1:500; Rabbit IgG), Cyclin D3 (1:1000; Mouse IgG) CDK 2 (1:1000; Rabbit IgG), CDK 4 (1:1000; Mouse IgG), GRP78/Bip (1:500; Rabbit IgG), IRE1 (1:1000; Rabbit IgG), p62 (1:500; Rabbit IgG), HDAC 1 and 2 (1:1000; Mouse IgG), HDAC 3 and 4 (1:1000; Rabbit IgG) and acetyl-histone H3 and H4 (1:1000; Rabbit IgG) were obtained from Cell Signaling Technologies (Beverly, MA, USA). Antibodies of ATF-6 (1:200; Mouse IgG), PERK (1:200; Mouse IgG), CHOP (1:150; Mouse IgG), LC3 (1:200; Mouse IgG) and AKT (ser473) (1:500; Rabbit IgG) were obtained from Santa Cruz Biotechnology (Santa Cruz, CA, USA). Anti-mouse, rabbit, and goat IgG secondary antibodies were obtained from Pierce (Rockford, IL, USA). Transfer membrane and ECL Western blotting substrate were purchased from Life Sciences (Amersham, UK). Fluo-3 AM and rhodamine 123 were purchased from Molecular Probes. The annexin-V-FITC/PI kit for detected cell apoptosis was purchased from Strong Biotech Corporation (Taipei, Taiwan).

### 4.2. MTT Anti-Proliferative Assay

Cell culture plates (96-well) were used for the MTT assay (thiazolyl blue tetrazolium bromide, Sigma-M2128). Seeding the cells at 5 × 10^4^ per well and treated with 25 and 50 μg/mL of EEAC and 100 μL of MTT buffer were added to the plates for 4 h. The ELISA reader (Anthoslabtec Instrument, Salzburg, Austria) was used at Ex:570 and Em:620 nm [56]. The results were showed as a percentage of the control ± SD from *n* = 4 wells in each experiment.

### 4.3. Colony Formation Assay

Cells were seeded at 5 × 10^3^ in the six-well plates before the treatment with EEAC (25 and 50 μg/mL) or 0.1% DMSO for 6 h and the medium was changed every three days for 14 days. After 14 days of incubation, colonies were stained with crystal violet (0.05% *w*/*v*) and counted under a microscope.

### 4.4. Annexin V/PI Assay for the Detection of Cell Apoptosis

The 10-cm dish was used for seeded cells at 5 × 10^5^ before treatment with 25 and 50 μg/mL EEAC or 0.1% DMSO for 48 h. Cells were collected in 100 μL binding buffer containing annexin V-FITC (10 μg/mL) and PI (50 μg/mL) for 15 min [56]. Cell were diluted to 2 × 10^5^ cells/mL using binding buffer before flow cytometer (Beckman Coulter, Taipei, Taiwan) detection. The results were analyzed with WinMDI software.

### 4.5. Determination of Mitochondrial Membrane Potential (MMP) Disruption and Calcium Concentration

Cells were seeded at 5 × 10^5^ in the 10-cm dish before the treatment with EEAC (25 and 50 μg/mL) or 0.1% DMSO for 48 h. Mitochondrial membrane potential disruption was detected with rhodamine 123 (1 μg/mL) [57], and calcium concentration was detected with Fluo-3 AM (1 μg/mL) [58] for 30 min using a flow cytometer and analyzed with WinMDI software. 

### 4.6. Determination and Analysis of Cell Cycle Population

Cells were seeded at 5 × 10^5^ in the 10-cm dish before the treatment with EEAC (25 and 50 μg/mL) or 0.1% DMSO for 48 h. Cells were collected and resuspended in PBS (150 μL) and 95% EtOH (375 μL) to fix the cells. After one day of incubation, the cells were subjected to permeabilization using 0.2% Triton X-100 in PBS and RNAase for 1 h. Cells were stained with PI (500 μg/mL) and the effect was detected by a flow cytometer and analyzed with Multicycle AV™ software.

### 4.7. Western Blotting Assay

Cells were lysates using ice cold RIPA for 24 h then centrifuged at 14,000 rpm for 30 min and the supernatant was collected to detect protein concentration using the BCA protein assay kit (Rockford, IL, USA). Twenty microliters of proteins were analyzed with 10% or 15% SDS gel electrophoresis and then transferred to PVDF membrane and 5% non-fat dry milk tTBS buffer was used for blocking for 1 h. Specific antibodies in 5% non-fat dry milk tTBS were used to detect protein expression for at least 2 h by immunoblotting and the membranes were detected by chemiluminescence kit (Rockford, IL, USA) [59].

### 4.8. HDAC Inhibition Activity Assay

Histone deacetylases inhibition activity was determined following the manufacturer’s protocol (BioVision cat: K331) [60]. The acetylated lysine side chain HDAC substrate was incubated with 25 and 50 μg/mL of EEAC containing HeLa nuclear extract for at least 30 min at 37 °C. Lysine was used to stop the reaction for 30 min and the inhibition was determined by excitation at 360 and emission at 450 nm using a spectrophotometer (Biotec synergy, Vermont, USA).

### 4.9. Animal Xenograft Model

Six-week-old male Balb/c nude mice were purchased from the National Laboratory Animal and Research Center (Taipei, Taiwan). T47D cells (1 × 10^6^) were resuspend in 200 μL PBS and injected into the right flank of each mouse subcutaneously for fourteen days and separated randomly into two groups (each group contained five mice). The EEAC (100 μg/g) or DMSO (100 μL) was intraperitoneally administered three times per week for 87 days and the size of the tumor spherical was measured by caliper as well as calculated with a formula: width^2^ × length/2. Mice were sacrificed using carbon dioxide. Our study was approved by the Animal Care and Treatment Committee of Kaohsiung Medical University (IACUC Permit Number 101136). All applicable international, national, and/or institutional guidelines for the care and use of animals were followed [59]. Our study was approved by the Ethics Committee of Kaohsiung Medical University (IACUC Permit Number 101136).

### 4.10. Immunofluorescence Analysis

The cells were fixed by 2% paraformaldehyde for 30 min and permeabilization was done using 0.2% Trition X-100 in PBS. Trition X-100 (T-PBS) containing 5% BSA was used to bind non-specific protein. Cells were incubated with the acetyl-histone H3, H4, GRP78/bip, and CHOP for 2 h and secondary antibodies for 1 h in a ratio of 1:1000 (Alexa Fluor 586-conjugated goat anti-mouse/rabbit IgG or Alexa Fluor 488-conjugated goat anti-mouse/rabbit IgG, Life Technologies, Carlsbad, CA, USA) at normal temperature. The PBS was washed twice and monitored with FV1000 confocal laser scanning microscope (Olympus, Tokyo, Japan) [59].

### 4.11. EEAC Preparation Procedures

The dish-cultured AC (EEAC) sample was provided by Oasis Bio-Tech Co., Ltd., New Taipei City, Taiwan. The ethanol extract of AC dish-cultured product (EEAC) was prepared as reported previously [55]. The dish cultured mushroom was refluxed with ethanol at 75 °C in a 1:10 (*w*/*v*) ratio for 2 h and the extract was storage for precipitate at 4 °C overnight and supernatant was filtered before further determination.

### 4.12. HPLC Conditions for EEAC Analysis

The EEAC analysis was executed on an LC-20A VP HPLC system (Shimadzu Inc.) which contained a quaternary pump (LC-20AT), an on-line degasser (DGU-14A), an autosampler (SIL-20AD), a photodiode-array detector (SPD-M20A) and a Class VP for data collection. The reverse-phase column chromatography was applied by an Agilent Poroshell 120 EC-C18 column (150 mm × 4.6 mm, i.d., 2.7 μm, Agilent Technologies, Palo Alto, CA, USA). The injection volume of the sample was 10 μL. The mobile phase composed of H_2_O containing 0.1% acetic acid (A) and ACN (B). A gradient program was used as follows: The initial elution condition was A:B (61:39, *v*/*v*), linearly changed to A:B (56:44, *v*/*v*) at 15 min, A:B (55:45, *v*/*v*) at 17.5 min, A:B (53:47, *v*/*v*) at 22.5 min, A:B (50:50, *v*/*v*) at 27.5 min, A:B (47:53, *v*/*v*) at 30 min, A:B (45:55, *v*/*v*) at 35 min, A:B (35:65, *v*/*v*) at 45 min, A:B (2:98, *v*/*v*) at 55 min, then ramped up to 100% B in 5 min, and finally decreased to 39% B in 1 min and held for 9 min, for regeneration. The solvent of the mobile phase was percolated through a 0.22 μm Millipore filter and degassed before entering the HPLC equipment. The flow rate was set up at 1.3 mL/min, the column temperature was retained at room temperature, and the detection wavelength was set up at 254 nm. The injecting sample was consisted of 1 mg EEAC dry extract which dissolving in 1 mL of methanol then filtered through a 0.45 μm membrane filter before entering the HPLC equipment.

### 4.13. Statistics

These results were expressed as mean ± SD. Comparison in each experiment was performed by Student’s *t*-test and two-way ANOVA for MTT results. *p* < 0.05 was considered significant. * *p* < 0.05; ** *p* < 0.01; *** *p* < 0.001.

## Supplementary Materials

Supplementary materials can be found at http://www.mdpi.com/1422-0067/20/4/833/s1.

## Abbreviations

AC	*Antrodia cinnamomea*
EEAC	Ethanol extract of artificially cultured *Antrodia cinnamomea*
ER	Endoplasmic reticulum
HPLC	High performance liquid chromatography
HAT	Acetyl transferases
HDAC	Histone deacetylases
UPR	Unfolded protein response
PERK	Pancreatic endoplasmic reticulum kinase
IRE1α	Inositol-requiring enzyme 1α
ATF6	Activating transcription factor 6
CDK	Cyclin-dependent kinases
SF	Submerged fermentation
SSC	Solid support culture
CWC	Cutting wood culture
DC	Dish culture
CHOP	CCAAT-enhancer-binding protein homologous protein
