# Supplementary material for: Antrodia cinnamomea, a Treasured Medicinal Mushroom, Induces Growth Arrest in Breast Cancer Cells, T47D Cells: New Mechanisms Emerge
Source: Int J Mol Sci. 2019 Feb 15;20(4):833. doi: 10.3390/ijms20040833 (PMC6412332; doi:10.3390/ijms20040833)
Supplement: Supplementary file 1 [file ijms-20-00833-s001.pdf]

Antrodia cinnamomea, a Treasured Medicinal Mushroom, Induces Growth Arrest in Breast Cancer Cells, T47D Cells: New Mechanisms Emerge

Supplementary Materials:

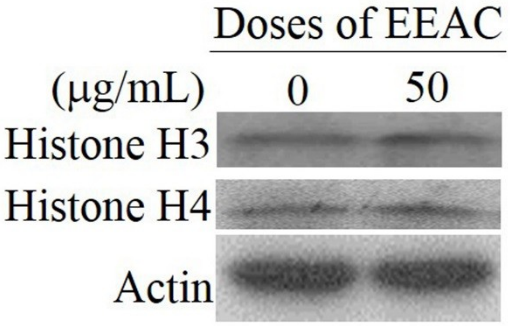

Figure S1. Expression of histone H3 and H4 protein after treated with EEAC for 48 h determined by Western blot assay. Actin was used as the loading control.
